# Assessment of heavy metals in total suspended particles and deposited dust in different areas in Assiut city, Egypt

**DOI:** 10.1038/s41598-025-18697-1

**Published:** 2025-09-17

**Authors:** Hazem A. Soliman, Ibrahim H. Saleh, Elsayed A. Shalaby, Zekry F. Ghatass, Ahmed R. Shatat, Mohamed Elsafi, Esraa H. Abdel-Gawad

**Affiliations:** 1 Environmental Studies Department, Institute of Graduate Studies and Research, Alexandria University, Alexandria, Egypt; 2 Chemistry Department, Faculty of Science, Al-Azhar University, Assiut, Egypt; 3 Physics Department, Faculty of Science, Alexandria University, Alexandria, Egypt

**Keywords:** Air pollution, Heavy metals, Total suspended particulate, Deposited dust, Health risk assessment, Environmental chemistry, Environmental chemistry

## Abstract

Heavy metals are major environmental pollutants due to their carcinogenic and toxic properties. This study investigates the levels of heavy metals different heavy metals in deposited dust and total suspended particles (TSP) in Assiut City and evaluates their potential health risks. The TSP and deposited dust samples were collected from six sites chosen based on their anthropogenic activities. The levels of the heavy metals in the collected samples were determined by an Inductively Coupled Plasma Optical Emission Spectrometer. During the seasons of the year, the average TSP concentrations in the most polluted location reached 1605 µg/m^3^, exceeding the air quality limits (230 µg/m^3^). Also, the most polluted locations went beyond the threshold value limit (TVL = 18 g/m^2^.month) in deposited dust rates. The average concentrations of heavy metals at selected sites ranged from 0.006 to 0.826 µg/m^3^ in TSP, and ranged from 6.302 to 64.65 µg/g in settled dust. These values were greater than those reported in comparable investigations conducted in other countries. By conducting a preliminary health risk assessment to the chronic exposure of the reported heavy metals concentrations, it was percussive that 20,000 cases per million are expected to get cancer because of the high-risk value of Chromium in the study area. The high Hazard Quotient values (ranging from 4 to 8) in this study suggest multi-organ toxicity, particularly to the nervous, reproductive, and respiratory systems. As a result, air quality management and the implementation of targeted policies within a risk management plan are necessary to lower the concentration of various heavy metals in the environment of this society.

## Introduction

Air pollution refers to harmful or excessive compounds present in the air that can affect human health and the environment. Air pollution is considered one of the most pressing environmental issues of the twenty-first century, primarily due to increasing industrial activities and energy production emissions^1^. Air pollution has also been linked to serious health cases such as cardiovascular, immunological, and neurological disorders besides certain cancers, prompting many countries to enforce stricter emission regulations^2^.

Among the various pollutants, particulate matter (PM) is one of the most dangerous air contaminants that endangers human health. PM is classified into fine particulate matter (PM_2.5_) with diameters of less than 2.5 μm and coarse particulate matter (PM_10_) with diameters of less than 10 μm^3^. These particles originate from a variety of sources, including industrial practices, vehicle exhaust, waste incineration, and fossil fuel combustion. Assessment of atmospheric particulate matter concentrations and related dangerous components is necessary for air quality management and epidemiological research^4^. Even at low concentrations, particulate matter pollution has a significant influence on human health. Premature mortality, lung cancer, and asthma attacks are just a few of the health issues that can come from an excess of particulate matter inhalation. Because of the severe effects resulting from particle matter, they contribute considerably to the global illness burden; hence, efforts to lower particulate matter concentrations must be sustained^5^.

In addition to airborne particles, deposited dust, which comprises particles with more than 10 μm, represents another environmental concern. It is easily settled from the atmosphere, posing a serious hazard to soil quality and atmospheric processes. The accumulated amount of deposited dust is affected by both meteorological conditions (wind speed, humidity, precipitation, and temperature) and anthropogenic activities such as transportation, fossil fuel burning, and numerous industrial operations^6^.

A critical aspect of particulate matter and deposited dust pollution is the presence of heavy metals bound to their surface. Heavy metals can accumulate in soil through deposition from the atmosphere, which can disrupt soil ecosystems and affect plant growth. They can also leach into water bodies and enter the food chain, leading to long-term environmental degradation and health risks. Furthermore, heavy metals absorbed by total suspended particles are entirely harmful when inhaled by humans^7^. Because of bioaccumulation, heavy metals in soil have detrimental repercussions for Living organisms. Unlike organic materials, heavy metals are not biodegradable and can only change their oxidation state, and their half-life in nature is more than 20 years^8^. Heavy metals have several toxicological consequences that have been extensively studied and recorded when taken in excess of the recommended daily diet. The quality and safety of food are impacted by high heavy metals concentrations in agricultural soils, which also raises worries about the risk of cancer, neurological damage, leukemia, mental illness, and other hazardous effects^9^.

Given these concerns, this study focuses on measuring heavy metals levels in total suspended particles and the deposited dust in selected sites in Assiut City for the aim of conducting regular health risk assessments to mitigate the impact of heavy metals contained in total suspended particles and deposited dust on human health and the environment. The results included in this investigation provide a baseline for future environmental monitoring as a step to mitigate the risks associated with total suspended particulates and deposited dust pollution.

## Materials & methods

### Study area

The research was conducted at various locations inside Assiut City. Assiut Governorate serves as the capital of Upper Egypt, situated 375 km south of Cairo (N 27°09’44.6” E 31°12’25.0” & N 27°11’7.76” E 31°4’32.81”). Many industrial facilities, including phosphate plants, thermal power stations, petroleum refineries, and cement factories are located around the city.

#### Meteorological parameters

**Fig. 1:**
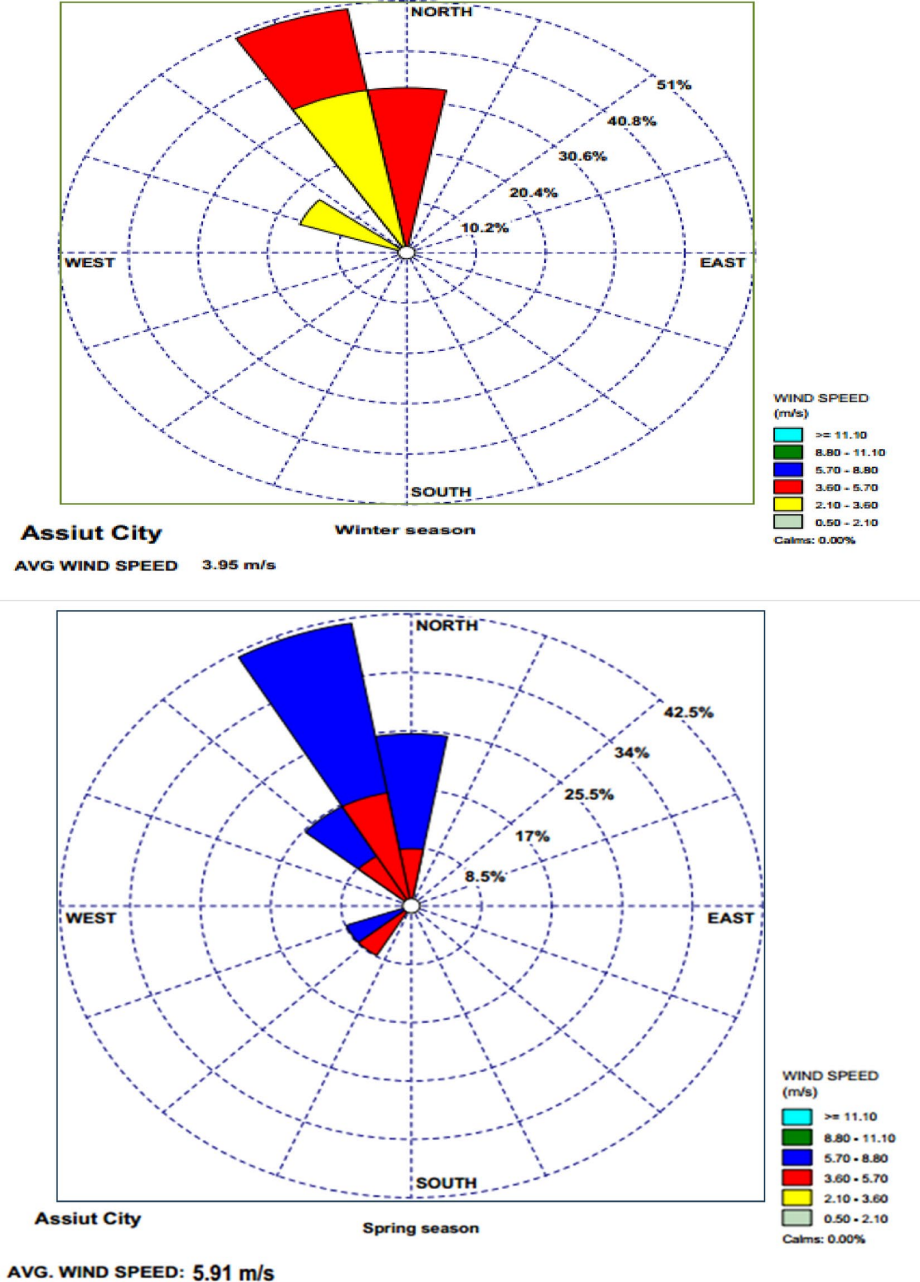

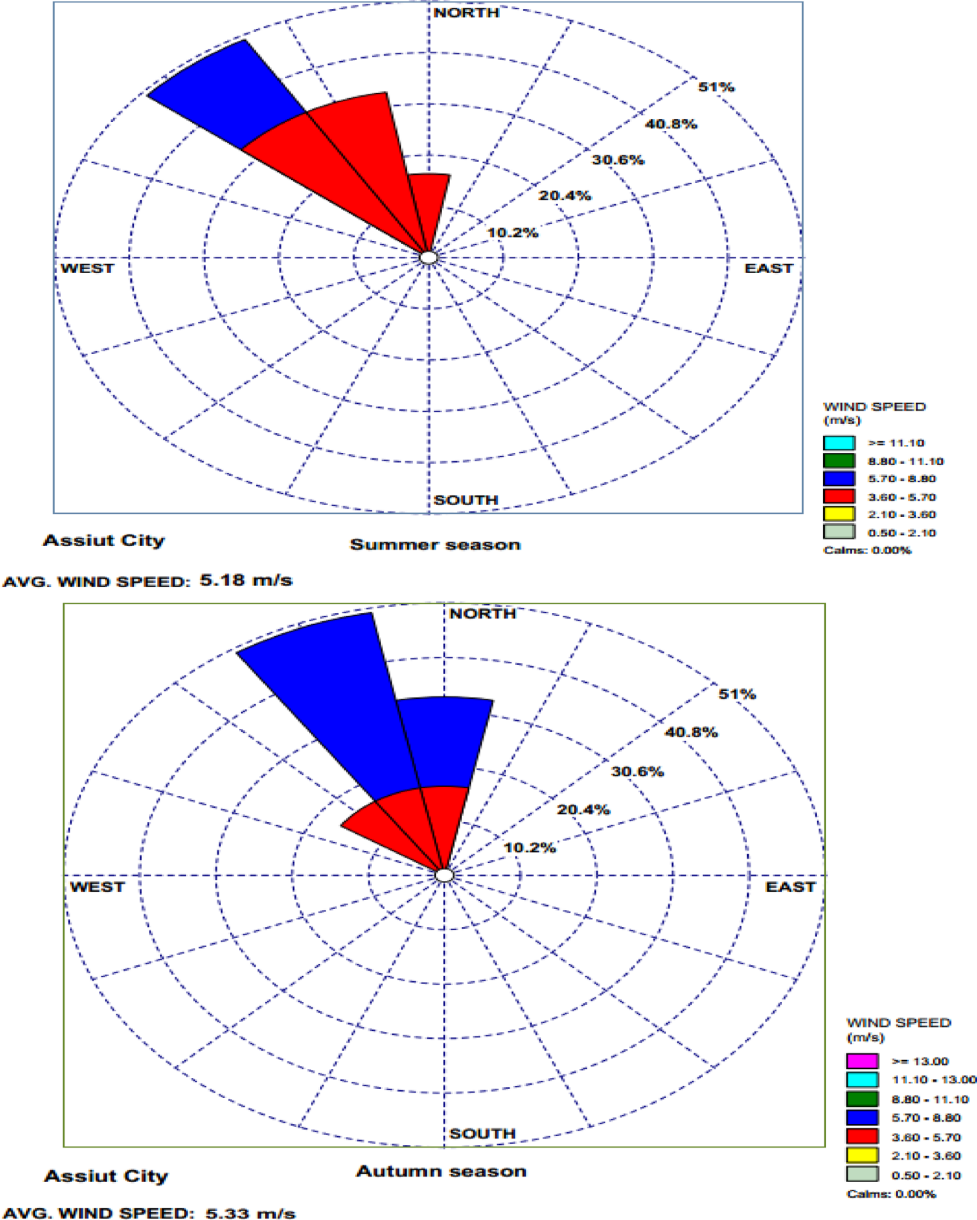
Wind rose for winter, spring, summer and autumn seasons in Assiut city.

Assiut Governorate’s weather is characterized by long and hot summers, cold winters, and low rainfall. Sandstorms are rare, but most Likely to occur in the spring. The Daily temperature ranged from 13.71 °C to 15.93 °C in winter, 18.99 °C to 29.82 °C in spring, 29.94 °C to 39.26 °C in summer, and 20.56 °C to 28.24 °C in autumn. The Daily relative humidity ranged from 56 to 59% (with a mean value of 58%) in winter, 30–46% (with a mean value of 37%) in spring, 29–30% (with a mean value of 29%) in summer, and 41–53% (with a mean value of 46%) in autumn, as shown in Table 1. In Figure 1, The prevailing wind directions were from northwesterly to northerly throughout the four seasons. The average wind speeds were 4.04 m/s in winter, 5.00 m/s in spring, 4.49 m/s in summer and 3.98 m/s in autumn. Meteorological parameters play a significant role in affecting the amount of deposited dust and total suspended particulates (TSP)^10–12^.

**Table 1:** Meteorological conditions in Assiut City during the study period.

Time	Temperature (°C)	Humidity (%)	Wind speed (m/s)
Dec-20	15.93	56	3.97
Jan-21	13.71	61	3.69
Feb-21	14.35	59	4.47
Mar-21	18.99	46	5.22
Apr-21	23.59	36	4.77
May-21	29.82	30	5.03
Jun-21	29.94	30	5.36
Jul-21	32.09	29	4.08
Aug-21	31.8	30	4.03
Sep-21	28.24	41	5.06
Oct-21	24.49	46	4.14
Nov-21	20.56	53	2.75
Winter	14.66	58	4.04
Spring	24.13	37	5.00
Summer	31.27	30	4.49
Autumn	24.43	46	3.98

### Samples collection

**Fig. 2:**
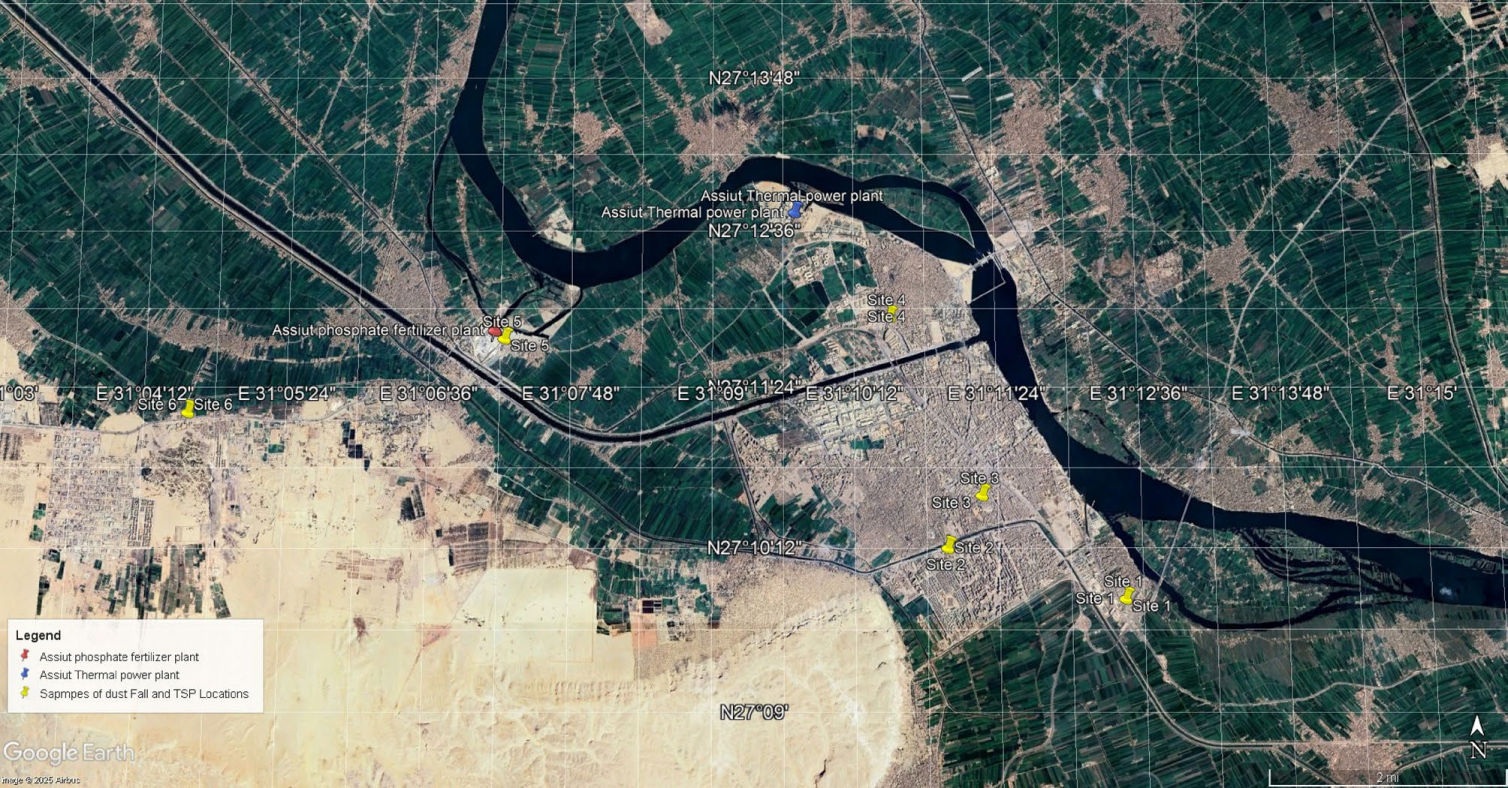
Locations of the collected samples at selected sites around Assiut city generated using satellite imagery from Google Earth^13^.

The total suspended particles samples and the deposited dust samples were collected from six sites (Al Hamraa, Al Arbaeen, Gisr Al Soltan, Al Wilidiyyah, Manqbad, Al Burah), which were selected based on anthropogenic activities (mining activities, fossil fuel combustion in power plants, highway roads, and heavy traffic) in Assiut City, as shown in Table 2 and Figure 2^13^. The duration of the study was one year, and the samples were taken each month. One TSP sample and one deposited dust sample were taken from each site per month. Within a year, 12 TSP samples and 12 deposited dust samples were taken from each location. For the selected six samples, 144 samples (6$\:\times\:$[12 + 12]) were collected in one year. The samples that were taken from sites 1, 2, 3, 4, and 5 were downwind phosphate plant & thermal power plant, while the sample that was taken from site 6 was upwind phosphate plant and thermal power plant. The geographical location of each site was determined by using a handheld global positioning system (GARMIN).

**Table 2:** Geographical locations of deposited dust and total suspended particulate samples in the study area.

Location	Coordinates	Location	Distance from the contamination source
SITE (1)	N 27°09’44.6” E 31°12’25.0”	Al Hamraa is located beside the highway linking Cairo and Aswan.	6 km from the thermal power facility, 9 km from the phosphate manufacturing plant.
SITE (2)	N 27°10’07.0” E 31°10’56.6”	Al Arbaeen is located beside car stations and roadway.	5 km from the thermal power facility, 7.2 km from the phosphate facility.
SITE (3)	N 27°10’30.6” E 31°11’14.30”	Gisr Al Soltan is located in the middle of Assiut city with heavy traffic.	4 km from the thermal power station, 7 km from the phosphate facility.
SITE (4)	N 27°11’51.05” E 31°10’28.40”	Al Wilidiyyah is located in the middle of Assiut City (downwind Assiut thermal power plant).	1.5 km from the thermal power station, 5 km from the phosphate facility.
SITE (5)	N 27°11’41.15” E 31° 7’12.37”	Mnaqbad is located downwind Assiut phosphate plant.	50 m from the phosphate facility, 4 km from the thermal power station
SITE (6)	N 27°11’7.76” E 31° 4’32.81”	Al Burah is located north of Assiut city (upwind Assiut phosphate plant & thermal power plant).	3 km from the thermal power station and 6 km from the phosphate facility.

#### Deposited dust samples

The deposited dust samples were collected monthly from all sites using plastic buckets with windshields based on the American Standard Test Method^14–16^. The buckets with a diameter (d) of 0.295 m were fixed at sampling sites at heights ranging from 2 to 3 m above the ground during the study period. After one month, the six buckets were taken from the sampling device, which contained deposited samples. The samples were closed tightly and transported to the laboratory to be used in experiments. The monthly deposited dust rate was calculated according to the ratio W/A, where W is the weight of dust in grams, and A is the cross area of the bucket in m^2^ $\:(A = \frac{{\pi \:}}{4}d^{2} )$.

The threshold limit values of deposited dust in different areas according to the limit for common pollutants^17^ are shown in Table 3.

**Table 3:** Threshold limit values of deposited dust^17^.

Band no.	Label	Deposited dust rate (D) (g/m^2^.month)	Action taken
1	Residential	D < 18	Allowed for residential and minor business use.
2	Industrial	24 < D < 36	Allowed for heavy industry and commercial use.
3	Action	36 < D < 72	Investigation and remediation are necessary if two consecutive months fall within this range or if more than three months occur within a year.
4	Alert	72 < D	Prompt action and remedation are necessary after the initial occurrence of the excess deposited dust rate; a report must be sent to the appropriate authority.

#### Total suspended particulates (TSP) samples

During the study period, TSP samples were taken once every season. Under typical working conditions, the samples were collected for 24 h using a low-volume sampler with a flow rate of 55 L/min and a filter paper Whatman GF/A glass microfibre filter with a 47 mm diameter^18^. The volume sampler was positioned 2 m to 3 m above the ground, oriented parallel to the ground, and installed downwind of the pollutant source. To acquire a representative sample, the device must not be situated beneath a tree, adjacent to a wall, or near other barriers that would impede unobstructed airflow from the surrounding atmosphere^19,20^. To mitigate the effects of humidity, which might affect weight, the filters were meticulously equilibrated in a desiccator before to and following sampling. The mass of TSP was then ascertained by reweighing the filters with a calibrated balance. Following a minimum of three weigh-ins for each filter, the net mass was determined by subtracting the pre-sampling weight from the post-sampling weight. Subsequent to weighing, the materials were preserved until digestion^21^. The concentration of TSP was ascertained using the subsequent equation:

1 $$TSP{\text{ }}\left( {\mu g/m^{3} } \right){\text{ }} = \frac{{\Delta w}}{{\Delta f~ \times ~\Delta t}}$$

where Δw (µg) is the difference in weight between the filter paper after and before sampling, Δf (m^3^/s) is the average flow rate [(initial flow rate + final flow rate)/2], and Δt (s) is the sampling time.

### Heavy metals measurements

**Fig. 3:**
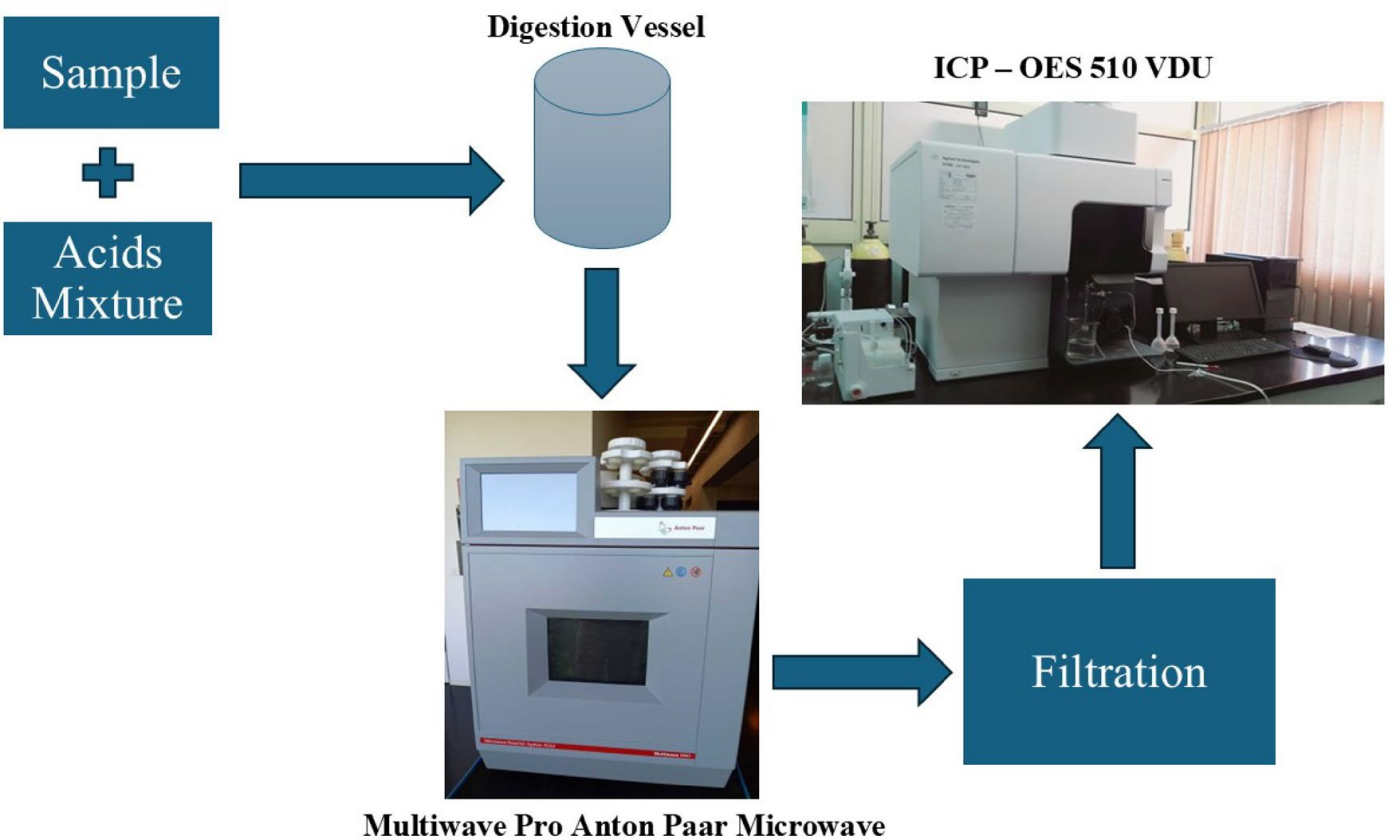
Procedure of Metal extraction from TSP and deposited dust samples.

**Fig. 4:**
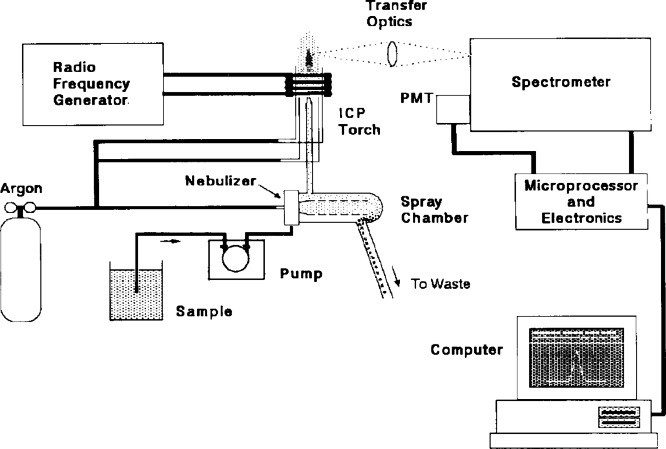
Block diagram of ICP- OES instrument^23^.

Arsenic (As), Cadmium (Cd), Cobalt (Co), Chromium (Cr), Nickel (Ni), Lead (Pb) and Vanadium (V) in deposited dust and total suspended particles samples have been measured by Inductively Coupled Plasma Optical Emission Spectrometer (ICP – OES 510 VDU) shown in Figure 3 and Figure 4. ICP-OES is allocated at the Plasma Emission Laboratory, Institute of Graduate Studies and Research, Alexandria University.

#### Sample digestion and preparation

Microwave-assisted acid digestion method was used to extract the heavy metals from the deposited dust and suspended particles samples. All samples were digested in a closed microwave digestion system (Multiwave Pro Anton Paar Microwave) according to the standard method of the Environmental Protection Agency (EPA)^22^. As illustrated in Figure 3, digestion is done in order to extract heavy metals from deposited dust and suspended particles samples. Each sample was placed in a Teflon digestion vessel with 3 mL of concentrated nitric acid (HNO_3_) and 7 mL of hydrochloric acid (HCL). All chemicals and reagents used were of high-purity analytical reagent grade. The sample after digestion was filtered using a volumetric flask and Whatman filter paper, then deionized water was added to complete the 50 ml volumetric flask. Before being used, distilled water and diluted (1:1) nitric acid were applied to all glassware and plastic containers. The target element stock solutions and all dilutions were prepared using double-distilled water. Using appropriate standard solutions made from the stock solutions, calibration curves were produced for every target element. The same procedure was used to extract and concentrate blank samples.

#### Theory of inductively coupled plasma optical emission spectrometry (ICP-OES)

The solutions that resulted after microwave acid digestion were analyzed using Inductively Coupled Plasma Optical Emission Spectrometry (ICP-OES), which is one of the most extensively used techniques for the approximation of compounds at atomic and molecular levels by determining their energy states by studying the light absorbed or emitted when they change states^23^. The sample solution is introduced into the system with high pressure, which is produced by pumps into a nebulizer, where the sample is converted into an aerosol. A spray chamber is between the nebulizer and the torch, which drains the excess droplets in aerosol through a drain and allows the sample to pass into the torch. In the torch, when plasma energy is given to an analysis sample from outside, the component elements (atoms) are excited. When the excited atoms return to a low energy position, emission rays (spectrum rays) are released, and the emission rays that correspond to the photon wavelength are collected by using various optics and wavelength devices are measured by using various detectors. The type of each element is determined by the position of the photon rays, while the content of each element is determined based on the intensity of the rays. Signal processing units and computers are present to read the result by the analyst. The operation conditions of Agilent ICP-OES 5100 VDV were: RF power = 1.2 KW, Plasma Flow = 12 L/min, Aux flow = 1 L/min, Nebulizer flow = 0.7 L/min, Read time (S) = 5 s, Sample Uptake time = 25 s, Replicate = 3 Times, Pump rate = 12 rpm, Stabilization time = 15 s. LOD (The limit of detection, which refers to the lowest concentration that can be distinguished) and LOQ (The limit of quantification, which refers to the lowest concentration that can be quantified with acceptable accuracy and precision) are presented in Table 4.

**Table 4:** Limit of detection (LOD) and limit of quantitation (LOQ).

No	Elements	Spike (µg/L)	Recovery %	LOD	LOQ
1	As 188.980 nm	500	109.6	17.61	52.85
2	Cd 214.439 nm	500	128	1.35	4.06
3	Co 230.786 nm	500	105.4	2.46	7.38
4	Cr 267.716 nm	500	144.2	14.28	42.85
5	Cu 327.395 nm	500	105.6	2.02	6.06
6	Mg 279.800 nm	500	141.4	8.29	24.87
7	Mn 257.610 nm	500	101.2	1.91	5.75
8	Mo 202.032 nm	500	102.2	6.18	18.55
9	Ni 231.604 nm	500	111.4	15.52	46.57
10	Pb 220.353 nm	500	104.2	9.72	29.18
11	Sb 206.834 nm	500	120.45	15.52	46.57
12	Se 196.026 nm	500	82.4	5.96	17.9
13	Sr 421.552 nm	500	95.2	0.773	2.31
14	Ti 334.941 nm	500	78.4	1.67	5.02
15	Zn 213.857 nm	500	104.6	10.17	30.51

#### Ambient air quality limits criteria

The ambient air quality limits for metals in TSP according to ONTARIO’s Ambient Air Quality Criteria^24^ and Egyptian air quality limit values^25^ are presented in Table 5 and Table 6.

**Table 5:** The ontario’s ambient air quality limits for metals in TSP.

Metal	Cd	Co	Cr	Ni	Pb	V	As
AQL (µg/m^3^)	0.025	0.1	0.05	0.015	0.5	2	0.3

**Table 6:** EEAA ambient air quality limits for metals in TSP.

Metal	Pb
AQL (µg/m^3^)	1.0

## Results and discussion

### Total suspended particulates (TSP)

#### Impact of site location on the average TSP concentration

**Fig. 5:**
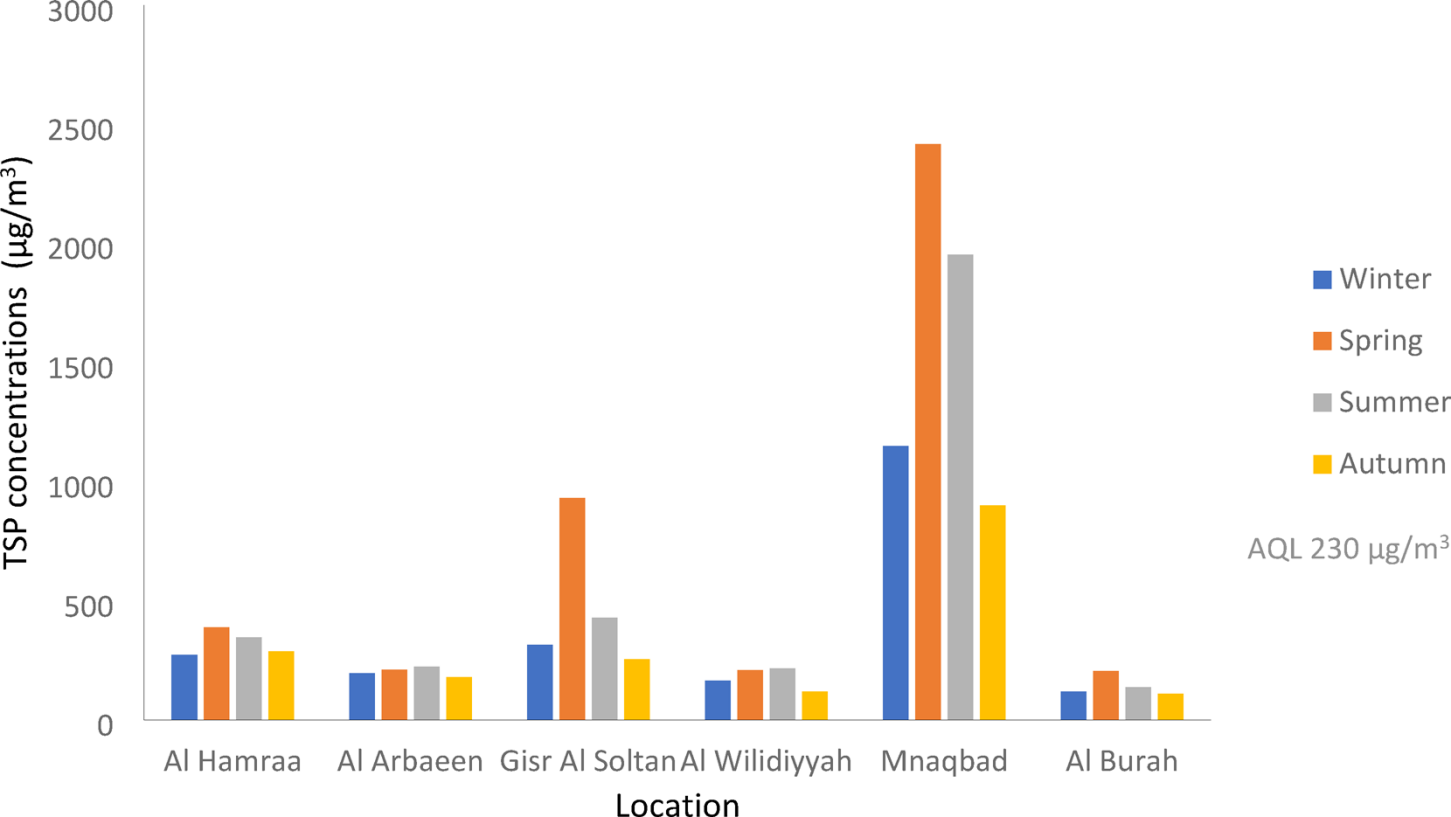
The variations of TSP concentrations in different sites in the study area during winter, spring, summer and autumn.

As shown in Figure 5 and Table 7 the Mnaqbad site in all seasons was found to be the worst site in the present study, where the average TSP concentration during the year was 1605 µg/m^3,^ which is greater than AQL (230 µg/m^3^)^25^ by 7 times. The highest TSP concentrations observed at the Mnaqbad site were attributed to its location, which is downwind of the fertilizer plant. Othman and Al-Masri^26^ reported the environmental impact of the fertilizer plant in the area. The air particulate emissions and dust are significant air quality problems in processing phosphate ore and packing phosphate fertilizers^27^.

Gisr Al Soltan site in all seasons was found to be the second worst site in the present study, where the average TSP concentration during the year was 483 µg/m^3,^ which is greater than AQL (230 µg/m^3^) by more than 2 times. The considerably high TSP concentration in the Gisr Al Soltan site is attributed to its location, which is downtown the city. Abbass and his colleagues^28^ reported that fine dust resuspension usually results from heavy traffic areas and vehicle emissions.

Al Hamraa site in all seasons was found to be the third worst site in the present study, where the average TSP concentration during the year was 324 µg/m^3^, which is greater than AQL (230 µg/m^3^) by 1.5 times. The high TSP concentration in the Al Hamraa site is attributed to its location, which is beside the highway that links Cairo and Aswan. As highway transportations increase, TSP pollutants increase on the roadside as a result of vehicle emissions, road dust, and rupture of tires.

During the seasons of the year, the average TSP concentrations in Al Arbaeen site, Al Wilidiyyah site, and Al Burah were 203 µg/m^3^, 177 µg/m^3^, and 143 µg/m^3^, respectively, and these values are lower than AQL (230 µg/m^3^).

**Table 7:** The average TSP concentration (µg/m^3^) in all sites in Assiut city.

Site/season	Al Hamraa	Al Arbaeen	Gisr Al Soltan	Al Wilidiyyah	Mnaqbad	Al Burah
Winter	273	196	316	165	1150	118
Spring	388	211	932	208	2417	206
Summer	346	224	429	216	1952	137
Autumn	288	179	254	118	900	109
Mean	324	203	483	177	1605	143
Standard Deviation	50.9	19.2	309.7	44.4	672.5	42.4
Standard Error	25.5	9.6	154.9	22.2	336.5	21.2
AQL	230 µg/m^3^					

#### Impact of seasonal variation on the average TSP concentration

**Fig. 6:**
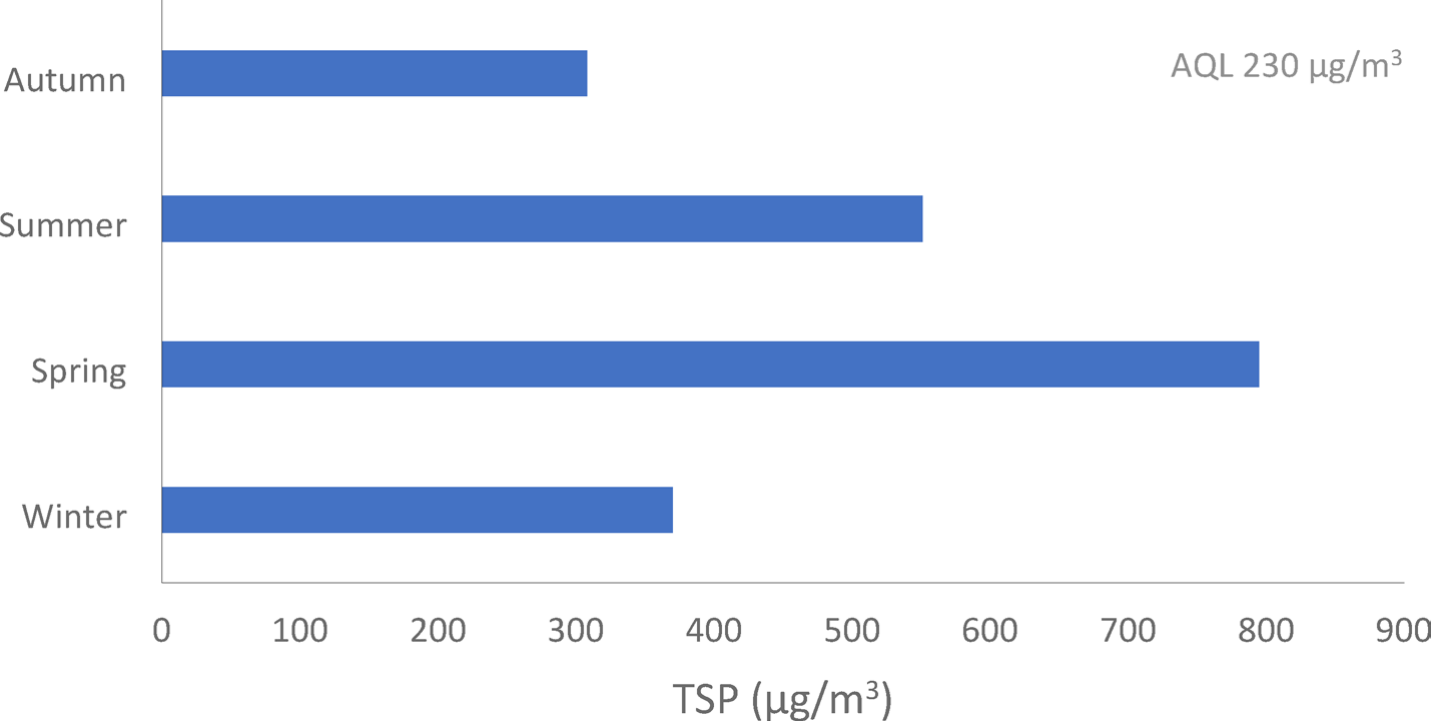
Seasonal variations of TSP average concentration in Assiut city.

As illustrated in Figure 6 and Table 8, the seasonal variations of TSP average concentration according to the current study are ranked as Spring ˃ Summer ˃ Winter ˃ Autumn, and this could be due to seasonality and reliance on meteorological parameters characterized by large amplitudes caused by changes in weather. The cause of the highest TSP concentration in the spring season as reported by Abed et al.^29^ is the blowing of the Khamasin wind during the spring season results in carrying dust from the western desert. This is supported by a previous study in Egypt that was reported by Hindy et al.^30^. Also, dry environmental conditions in the springtime can create higher suspended particles from the roadway and soil surfaces otherwise, the wind speed and direction impact TSP concentration, and dry air extends the lifespan of fine particulates in the ambient air, resulting in greater TSP concentrations in the air^31^.

**Table 8:** Seasonal variations of TSP average concentration (µg/m^3^) in Assiut city.

Site/season	Winter	Spring	Summer	Autumn
Al Hamraa	273	388	346	288
Al Arbaeen	196	211	224	179
Gisr Al Soltan	316	932	429	254
Al Wilidiyyah	165	208	216	118
Mnaqbad	1150	2417	1952	900
Al Burah	118	206	137	109
Mean	370.7	793.7	550.7	308.0
Standard deviation	372.4	873.7	873.3	316.5
Standard error	152	365.5	278.6	129.2
AQL	230/m^3^			

### Deposited dust

#### Impact of site location on the average the average deposited dust rates

**Fig. 7:**
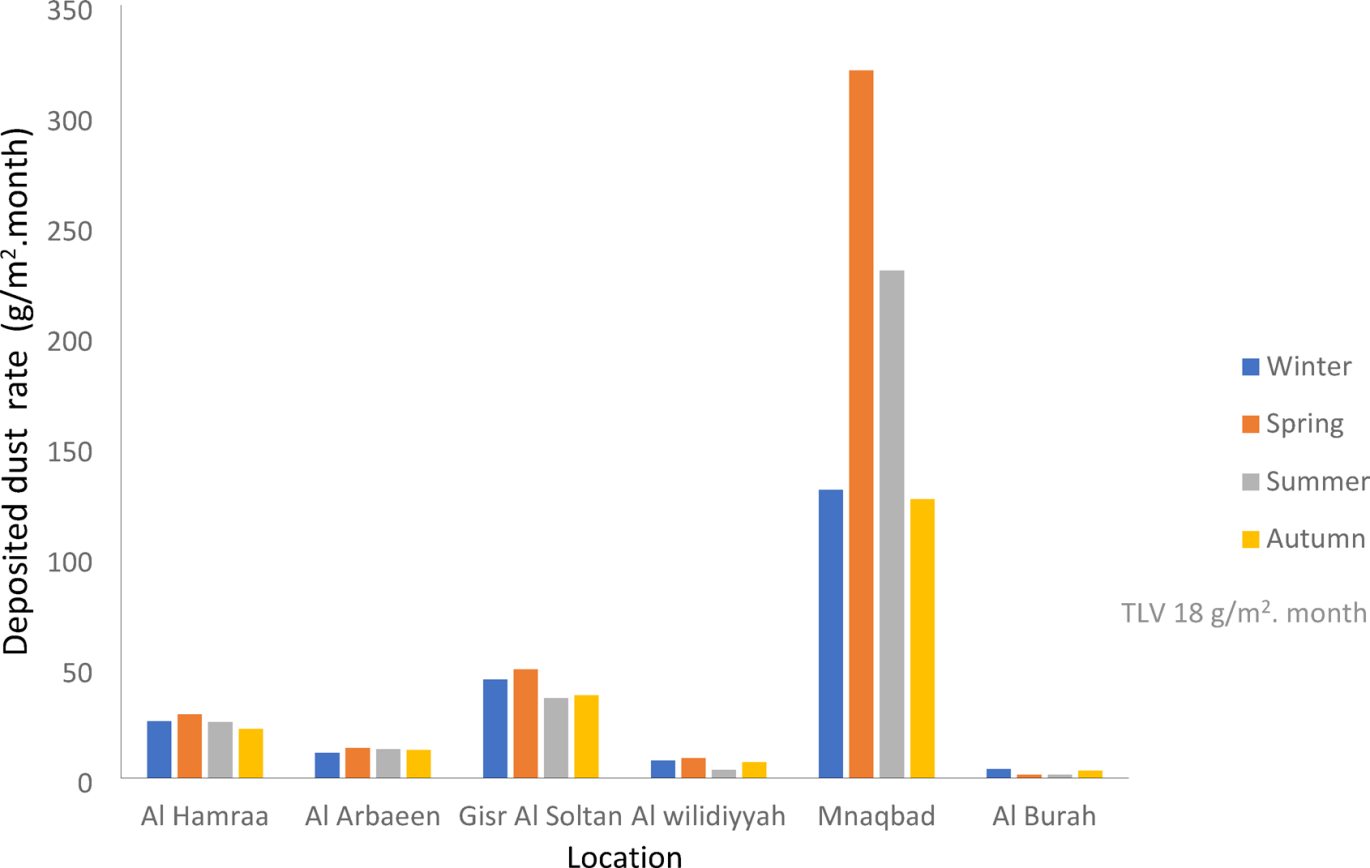
The variations of deposited dust rate in different sites in the study area during winter, spring, summer and autumn.

As shown in Figure 7 and Table 9 and the average deposited dust rates in all sites were ranked as Mnaqbad site ˃ Gisr Al Soltan site ˃ Al Hamraa site ˃ Al Arbaeen site ˃ Al Wilidiyyah site ˃ Al Burah site. Mnaqbad site in all seasons was found to be the first worst site in the present study where the average deposited dust rate was 201.7 g/m^2^.month which exceeded TLV (18 g/m^2^.month)^17^ by more than 11 times. According to SANS 1929^17^, if the dust deposition rate was more than 72 g/m^2^.month, it is classified as “Alert” and needs immediate action and remediation, which necessitates raising the incident report to the relevant authority. The highest deposition rates at the Mnaqbad site were attributed to its location, which is downwind of the fertilizer plant.

Gisr Al Soltan site in all seasons was observed as the second worst site where the average deposited dust rate was 41.8 g/m^2^.month exceeding the TLV (18 g/m^2^.month) by more than 2.3 times. According to SANS 1929^17^, if the dust deposition rate was from 36 < D < 72 g/m^2^.month it is classified as “Action” and requires investigation and remediation if two sequential months lie in this band or more than three occur in a year. The high deposition rate at the Gisr Al Soltan site is attributed to its location, which is downtown where heavy traffic and vehicle emissions exist around this area.

Al Hamraa Site in all seasons was observed as the third worst site where the average deposited dust rate was 25.5 g/m^2^.month exceeding the TLV (18 g/m^2^.month). According to SANS 1929^17^, if the deposition rate was from (24 < D < 36) g/m^2^.month it is classified as industrial, and this is permissible for commercial and industrial situations. The high dust deposition rates at the Al Hamraa site were attributed to its location beside the highway that links Cairo and Aswan, and other environmental impacts around this area^32^.

During the seasons of the year, the average deposited dust rates in Al Arbaeen site, Al Wilidiyyah site, and Al Burah were 12.6, 6.9, and 2.5 g/m^2^.month, respectively, and these values are lower than the normal limits of dust deposition rate (D < 18) g/m^2^.month according to SANS 1929^17^, this is classified as Residential, and this is permissible for residential and light commercial.

The impact of the phosphate plant and the heavy traffic in Assiut city made the mean settling rate of deposited dust reach 49 g/m^2^.month. According to SANS 1929^17^, the value of 36 < D < 72 g/m^2^/month is classified as actionable, which means that there is a need for investigation and remediation if two consecutive months or more than three occur in a year.

**Table 9:** The average of deposited dust rates (g/m^2^. month) in all sites in Assiut city.

Site/season	Al Hamraa	Al Arbaeen	Gisr Al Soltan	Al wilidiyyah	Mnaqbad	Al Burah
Winter	25.7	11.3	44.5	7.8	130.5	4.0
Spring	28.8	13.6	49.1	9.0	320.4	1.3
Summer	25.2	13.0	36.1	3.6	229.7	1.4
Autum	22.2	12.6	37.4	7.1	126.3	3.3
Mean	25.5	12.6	41.8	6.9	201.7	2.5
Standard deviation	2.7	1.0	6.1	2.3	92.4	1.4
Standard error	1.4	0.5	3.1	1.2	46.2	0.7
TLV	18 g/m^2^. Month					

The impact of the phosphate plant and the heavy traffic in Assiut city made the mean settling rate of deposited dust reach 49 g/m^2^.month. According to SANS 1929^17^, the value of 36 < D < 72 g/m^2^/month is classified as actionable, which means that there is a need for investigation and remediation if two consecutive months or more than three occur in a year.

**Fig. 8:**
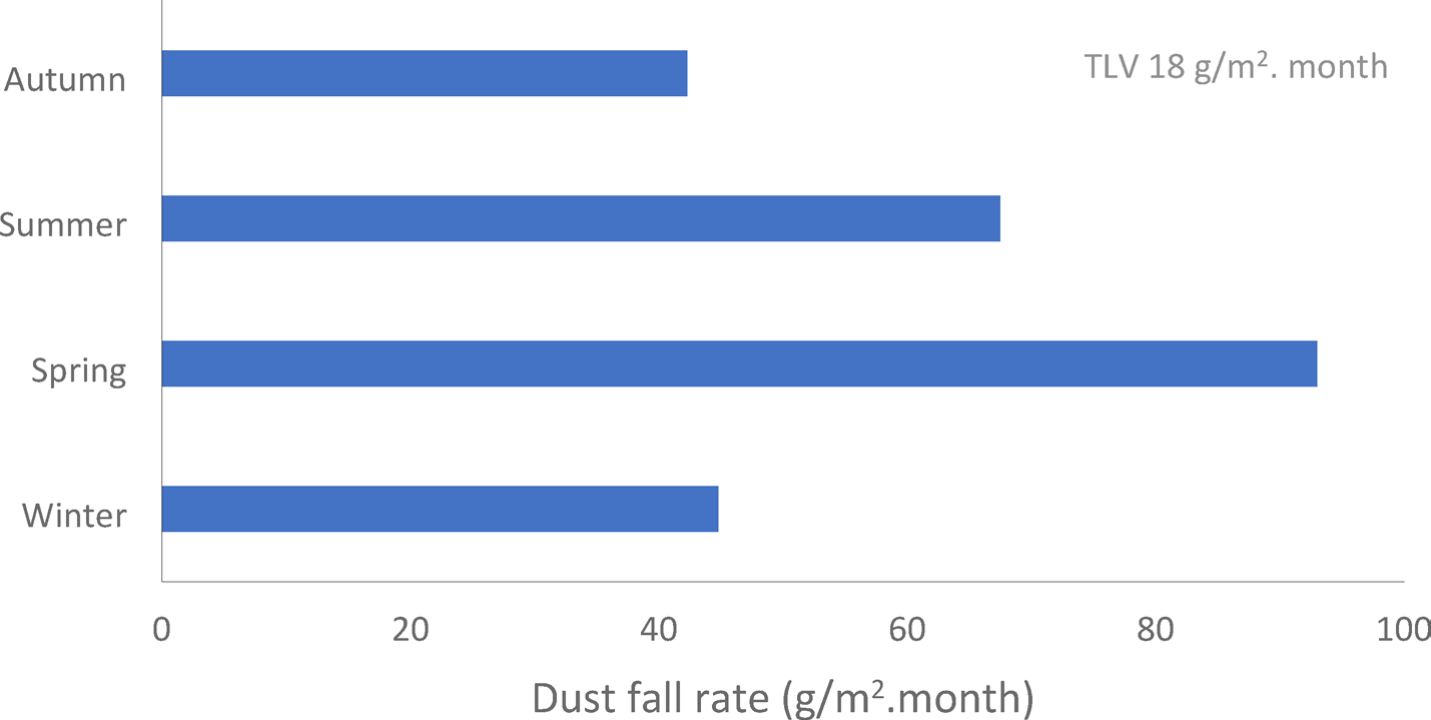
Seasonal variations of average deposited dust rate (g/m2. month) in Assiut city.

#### Impact of seasonal variation on the average deposited dust rates

As illustrated in Figure 8 and Table 10, the seasonal variations of average deposited dust rate according to the current study are ranked as spring ˃ summer ˃ winter ˃ autumn. The variations in deposited dust rates among the different seasons could be due to seasonality and reliance on meteorological parameters characterized by large amplitudes caused by changes in weather. The highest dust deposition rate in spring is due to high wind speed, and low humidity, while the lowest dust deposition rate in autumn is due to low wind speed^33^.

**Table 10:** Seasonal variations of average deposited dust rate (g/m^2^. month) in Assiut city.

Site/season	Winter	Spring	Summer	Autumn
Al Hamraa	25.7	28.8	25.2	22.2
Al Arbaeen	11.3	13.6	13	12.6
Gisr Al Soltan	44.5	49.1	36.1	37.4
Al Wilidiyyah	7.8	9	3.6	7.1
Mnaqbad	130.5	320.4	229.7	126.3
Al Burah	4	1.3	1.4	3.3
Mean	37.3	70.4	51.5	34.8
Standard deviation	48.0	123.7	88.3	46.5
Standard error	19.6	50.5	36.0	19.0
TLV	18 g/m^2^. month			

### Impact of meteorological parameters on pollutant concentrations

The direction of the wind (northwesterly to northerly winds) has influenced the dispersion of the pollutants. In Manqabad and Gisr Al Soltan, which were located downwind of many pollution sources such as the thermal power plant and phosphate plant, the concentration of TSP and deposited in these sites was higher than in other sites like Al Burah, which was located upwind. Moreover, high wind speed in spring (5 m/s) may be the reason behind the highest amount of TSP. The elevated temperature recorded during summer (31.27 °C) probably enhanced the vertical mixing and atmospheric turbulence. The elevated TSP in summer indicates the resuspension of deposited dust due to dry and hot conditions. The interplay between wind, temperature and humidity had a combined effect on the presence of pollutants in the atmosphere. For instance, while spring had the highest wind speeds and moderate temperatures, it also showed the highest concentrations of both TSP and deposited dust. This might be attributed to the enhanced dispersion of pollutants from pollution sources to the sites investigated, besides the limited humidity or precipitation to remove pollutants from the atmosphere^34^.

### Heavy metal concentrations

Samples of TSP and deposited dust from Manqabad site and Gisr Al Soltan site were collected to analyze the heavy metals content. We chose Mnaqbad and Gisr Al Soltan because it was observed that, during all seasons, both sites have the highest TSP concentrations and the highest deposited dust rates compared to the other sites under investigation. Moreover, Mnaqbad is located 50 m (minimum distance) downwind Assiut phosphate plant. Additionally, Gisr Al Soltan is located in the middle of Assiut City, and it is known by its heavy traffic and its location was very close to the thermal power plant. The concentrations of the heavy metals (As, Cd, Co, Cr, Ni, Pb and V) were measured using an ICP-OES spectrometer and the results will be presented and discussed in the section below.

#### Heavy metal concentrations in deposited dust

**Fig. 9:**
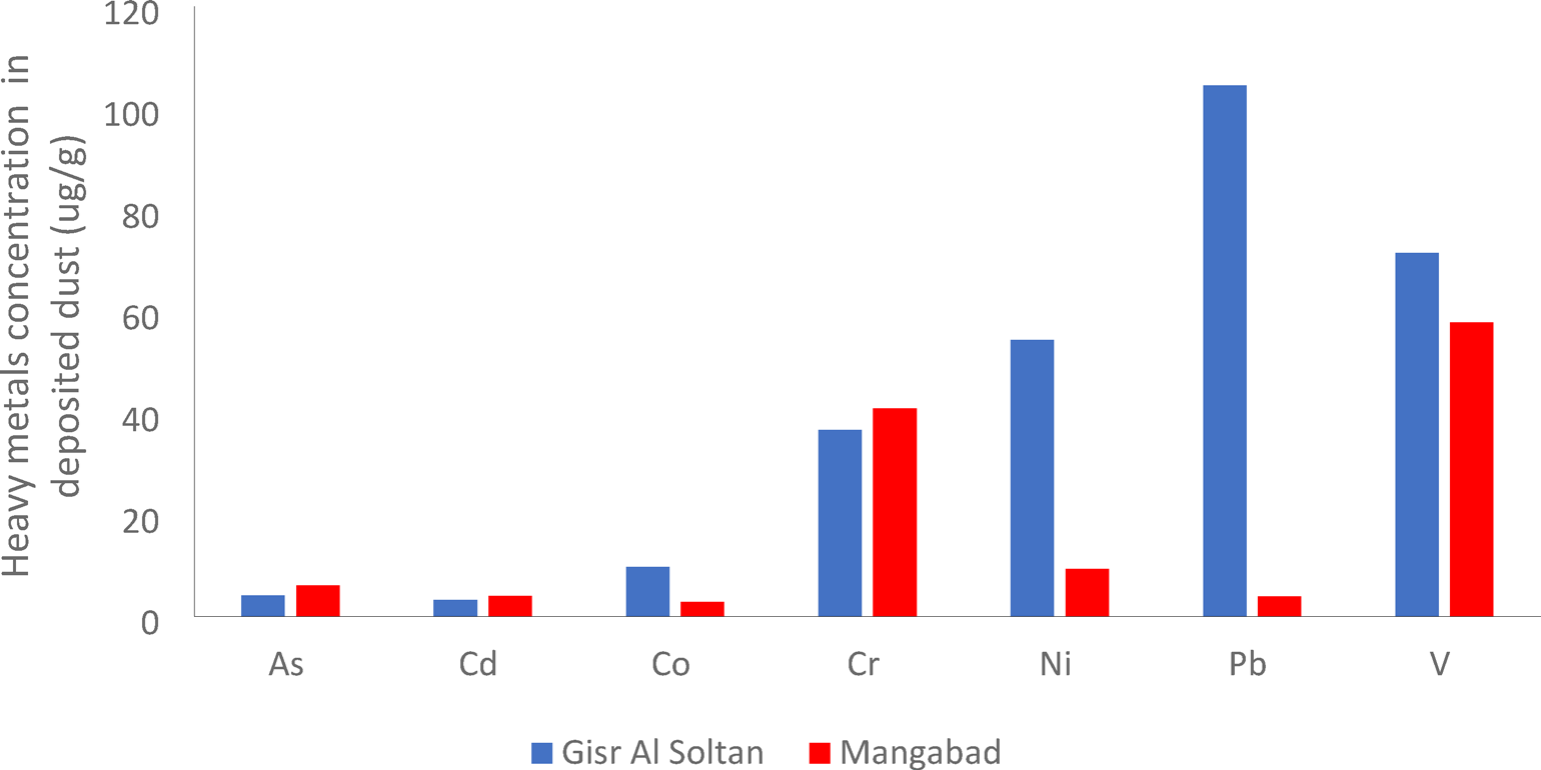
Average concentration of heavy metals concentrations in deposited dust samples at Gisr Al Soltan site and Manqabad site.

The concentrations of As, Cd, Co, Cr, Ni, Pb, and V in deposited dust samples in the current study at the Gisr Al Soltan site and Manqabad site were summarized in Figure 9 and Table 11.

**Table 11:** Min., max., and average concentration of heavy metals in deposited dust samples ($\:\mu\:$g/g) at the study area compared with other worldwide studies.

Element/location		As	Cd	Co	Cr	Ni	Pb	V	References
Gisr Al Soltan	Min	0.58	1.73	7.43	8.82	50.14	25.76	0.17	The present study
	Max	9.53	5.20	11.21	47.86	59.64	162.51	105.69	
	Average	4.10	3.26	9.76	36.72	54.30	104.42	71.49	
Manqabad	Min	0.92	0.20	0.21	0.19	0.79	0.20	2.58	
	Max	14.12	5.53	5.55	81.07	17.79	8.11	113.90	
	Average	6.14	4.05	2.88	40.95	9.34	3.94	57.82	
Egypt (Assuit) ± Standard deviation		5.12 ± 4.83	3.66 ± 2.02	6.32 ± 3.85	38.83 ± 31.84	31.82 ± 24.97	54.18 ± 50.90	64.65 ± 51.33	
China (Ebinur Lake Basin)		22.74	1.67	13.44	203.34	31.18	1841.33	58.20	^35^
Egypt (Alexandria)		-	0.33	2.7	24.3	14.4	70.3	-	^36^
Mongolia (Ulaanbaatar)		16.5	-	10.4	70.2	21.3	51.7	-	^37^
India (Kharagpur)		-	-	22.42	200.45	56	194.87	115.08	^38^
China (Beijing)		-	-	11.5	72.1	37.6	119	61.7	^39^
China (Shijiazhuang)		-	2.84	14.9	141.4	42.1	177	56.9	^40^
Tajikistan (Khujand)		17.5	-	6.8	92.5	44.6	73.6	62.2	^41^

The comparative analysis of heavy metals bound to deposited dust between Assiut City, Egypt and different countries worldwide (Table 11) shows the different regional pollution characteristics due to the variance in anthropogenic activities.

##### Arsenic (As)

Arsenic concentrations in deposited dust at Gisr Al Soltan site ranged from 0.58 µg/g to 9.53 µg/g and at the Manqabad site ranged from 0.92 µg/g to 14.12 µg/g. The average Arsenic concentration in Assiut City was 5.12 µg/g, and by comparing Arsenic concentration with those in other studies in different countries, it was clear that the levels of Arsenic in this study were lower than China (Ebinur Lake Basin), Mongolia (Ulaanbaatar), and Tajikistan (Khujand), showing moderate contamination levels. This difference may be attributed to the variation in geochemical baselines and different industrial activities in these countries.

##### Cadmium (Cd)

Cadmium concentrations in deposited dust at Gisr Al Soltan site ranged from 1.73 µg/g to 5.20 µg/g and at the Manqabad site ranged from 0.20 µg/g to 5.53 µg/g. Assiut City showed high Cd levels (3.66 µg/g) compared to Alexandria in Egypt and Shijiazhuang in China, which might be attributed to different industrial activities like waste incineration and phosphate fertilizers production.

##### Cobalt (Co)

Cobalt concentrations in deposited dust at Gisr Al Soltan site ranged from 7.43 µg/g to 11.21 µg/g and at the Manqabad site ranged from 0.79 µg/g to 17.79 µg/g. The average Co concentration in Assiut City (6.32 µg/g) was lower than in other industrial cities such as Beijing in China and Kharagpur in India, showing less pollution concerns in Assiut City.

##### Chromium (Cr)

Chromium concentrations in deposited dust at Gisr Al Soltan site ranged from 8.82 µg/g to 47.86 µg/g and at the Manqabad site ranged from 0.19 µg/g to 40.95 µg/g. The average Cr concentration in Assiut City (38.38 µg/g) is considered high, even if it is lower than in India and China. The high levels of Cr found in deposited dust in Assiut City may be attributed to the excessive industrial activities and vehicle abrasion, hence, it requires pollution management.

##### Nickel (Ni)

Nickel concentrations in deposited dust at Gisr Al Soltan site ranged from 50.14 µg/g to 59.64 µg/g and at the Manqabad site ranged from 0.79 µg/g to 17.79 µg/g. The average Ni concentration in Assiut City was 31.82 µg/g. By comparing Nickel concentration with those in other studies in different countries it was clear that the levels of Ni in this study were lower than India (Kharagpur), China (Beijing), China (Shijiazhuang) and Tajikistan (Khujand) with the same value compared to China (Ebinur Lake Basin); indicating limited nickel-based industrial activities in Assiut City.

##### Lead (Pb)

Lead concentrations in deposited dust at Gisr Al Soltan site ranged from 25.76 µg/g to 162.51 µg/g and at the Manqabad site ranged from 0.20 µg/g to 8.11 µg/g. The extremely high levels of Pb in Gisr Al Soltan compared to the lower levels in Manqabad indicate significant variance in urbanization and the use of leaded materials in different activities, which necessitate continuous monitoring and control of these activities to prevent Pb toxicity.

##### Vanadium (V)

Vanadium concentrations in deposited dust at the Gisr Al Soltan ranged from 0.17 µg/g to 105.69 µg/g and at the Manqabad site ranged from 2.58 µg/g to 113.90 µg/g. The average V concentration in Assiut City was 64.65 µg/g. When comparing Vanadium concentration with those in other studies in different countries, the levels of V in this study were higher than China (Ebinur Lake Basin), China (Beijing), China (Shijiazhuang) and Tajikistan (Khujand), showing excessive fossil fuel combustion.

From Table 11 and Figure 9, the concentrations of heavy metals in deposited dust have a decreasing order (Pb > Cr > Ni > Co > V > Cd > As), showing lead and chromium as the dominant pollutants, assuring the need for air quality management and public health strategies in Assiut City, Egypt.

#### Heavy metal concentrations in TSP

**Fig. 10:**
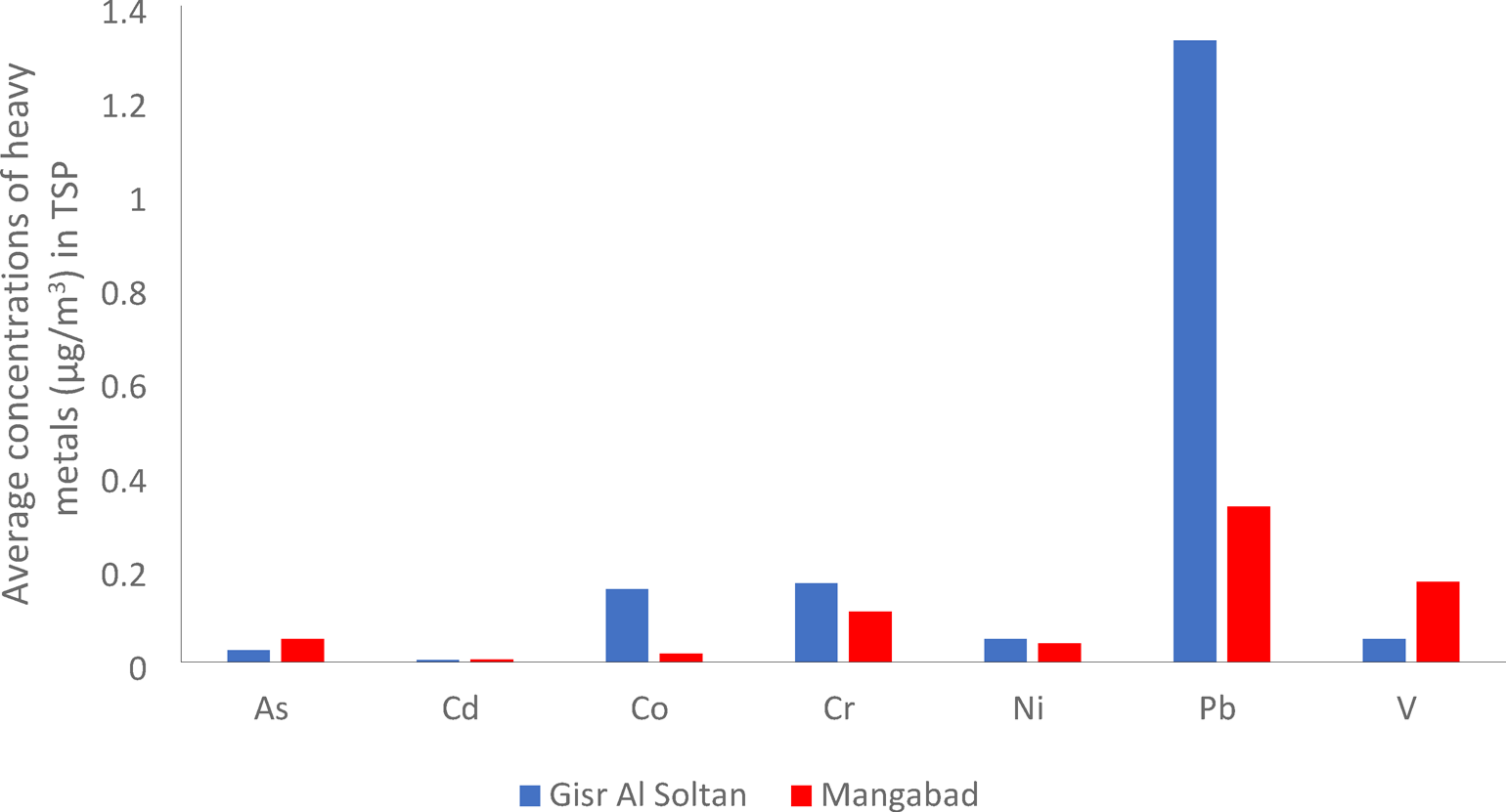
Average concentrations of heavy metals in TSP at Gisr Al Soltan site and Manqabad site.

The concentrations of As, Cd, Co, Cr, Ni, Pb, and V in TSP of samples collected from Gisr Al Soltan site and Manqabad site were summarized in Figure 10 and Table 12. The heavy metals released from industrial emissions, fossil fuel combustion, and automobile exhaust are rapidly absorbed by fine particulate matter, thus impacting air quality^42^.

**Table 12:** Min., max., and average concentrations of heavy metals (µg/m^3^) in TSP samples in this study compared to other studies.

Element/locations		As	Cd	Co	Cr	Ni	Pb	V	References
Gisr Al Soltan	Min	0.009	0.002	0.013	0.035	0.022	0.072	0.033	The present study
	Max	0.066	0.012	0.424	0.424	0.109	4.034	0.07	
	Average	0.026	0.005	0.156	0.168	0.049	1.326	0.05	
Manqabad	Min	0.007	0.002	0.01	0.038	0.017	0.071	0.029	
	Max	0.11	0.011	0.026	0.173	0.062	0.756	0.298	
	Average	0.049	0.006	0.018	0.108	0.040	0.332	0.172	
Egypt (Assuit) ± Standard deviation		0.038 ± 0.035	0.006 ± 0.004	0.087 ± 0.014	0.138 ± 0.12	0.044 ± 0.03	0.829 ± 0.56	0.111 ± 0.01	
Egypt (Port Said)		-	0.17	-	1.64	1.35	1.3	-	^43^
India (Bhubaneswar)		0.014	-	0.006	0.026	0.036	0.065	0.005	^44^
Saudi Arabia (Red Sea Coast)		0.002	0.0002	-	0.022	0.0122	0.0265	0.203	^45^
Saudi Arabia (Makkah)		0.50	0.02	-	0.02	0.43	0.08	0.01	^46^
China (Yangtze)		0.067	0.014	0.005	0.039	0.026	0.65	0.04	^47^
China (Guangzhou)		-	0.003	0.001	0.016	0.01	0.101	-	^21^
AQL		0.3	0.25	0.1	0.05	0.015	0.5	2	^24^
AQL		-	-	-	-	-	1	-	^25^

The comparative analysis of airborne heavy metals (Table 12) shows significant variation between the studied area (Assiut City in Egypt) and other locations worldwide. This is probably due to differences in urban planning, industrialization, vehicular emissions and other anthropogenic activities.

##### Arsenic (As)

Arsenic concentrations in TSP at Gisr Al Soltan site ranged from 0.009 µg/m^3^ to 0.066 µg/m^3^ and at Mangabad site ranged from 0.007 µg/m^3^ to 0.110 µg/m^3^. The average of Arsenic concentration in Assiut City was 0.038 µg/m^3,^ which fell below ONTARIO’ Ambient Air Quality Criteria (AQL) of 0.3 µg/m^3,^ indicating no public health risk. However, when comparing Arsenic concentration in Assiut city with its concentration in different countries, it was clear that the concentration of Arsenic in the present study was higher than the compares in India (Bhubaneswar) and Saudi Arabia (Red Sea Coast). This could reflect the origins of emissions in Assiut City, such as the thermal power plant and industrial operations. Moreover, the lower concentrations in those areas could be attributed to the strict air quality policy applied there.

##### Cadmium (Cd)

Cadmium concentrations in TSP at Gisr Al Soltan site ranged from 0.002 µg/m^3^ to 0.12 µg/m^3^ and at the Mangabad site ranged from 0.002 µg/m^3^ to 0.011 µg/m^3^. The average Cd levels in Assiut City (0.006 µg/m^3^) are considered low compared to Port Said (0.17 µg/m^3^), which highlights the regional variation within Egypt. When comparing Cd concentration with other studies in different countries, the lower concentrations in Assiut City compared to Saudi Arabia (Makkah) and China (Yangtze) may reflect fewer high-temperature industrial processes like smelting, which are major sources of Cd bound to total suspended matter. Additionally, Cd levels in Assiut City were below the AQL (0.25 µg/m^3^), suggesting low pollution concern.

##### Cobalt (Co)

Cobalt concentrations in TSP at Gisr Al Soltan site ranged from 0.013 µg/m^3^ to 0.424 µg/m^3^ and at the Mangabad site ranged from 0.010 µg/m^3^ to 0.026 µg/m^3^. The average Co concentration in Assiut City was (0.087 µg/m^3^), which is considered close to the AQL (0.1 µg/m^3^) but substantially higher than reported in India and China. The elevated level of Co increases the potential respiratory toxicity upon long-term exposure.

##### Chromium (Cr)

Chromium concentrations in TSP at Gisr Al Soltan site ranged from 0.035 µg/m^3^ to 0.424 µg/m^3^ and at Mangabad site ranged from 0.038 µg/m^3^ to 0.173 µg/m^3^. Cr average concentration in Assiut City (0.138 µg/m^3^) exceeds the AQL (0.05 µg/m^3^) and surpasses its comparable international values in other studies. This means a seriously polluted environment from the investigated point sources, which are the thermal power plant, phosphate fertilizer factory, and highway. The measured Cr levels raise environmental and health red flags, especially the carcinogenicity of hexavalent chromium.

##### Nickel (Ni)

Nickel concentrations in TSP at Gisr Al Soltan ranged from 0.022 µg/m^3^ to 0.109 µg/m^3^ and at Mangabad ranged from 0.017 µg/m^3^ to 0.062 µg/m^3^. The average Ni levels in Assiut City (0.045 µg/m^3^) exceed the AQL (0.015 µg/m^3^), highlighting high exposure risk. Despite the lower levels of Ni found in TSP in Assiut City in Egypt compared to Saudi Arabia, it remains higher than in many other countries, assuring the need for air pollution control in different industrial activities in Assiut City.

##### Lead (Pb)

Lead concentrations in TSP at Gisr Al Soltan site ranged from 0.072 µg/m^3^ to 4.034 µg/m^3^ and at Mangabad site ranged from 0.017 µg/m^3^ to 0.756 µg/m^3^. The average Pb concentration in Assiut City (0.829 µg/m^3^) raises concerns as it exceeds AQL (0.5 µg/m^3^) and international comparisons. Moreover, Pb levels in Assiut city are above Egypt’s national limit (1 µg/m^3^) according to Egyptian Environmental Law 4/94. Despite banning Pb from gasoline, residual sources as old paints, batteries, and industrial emissions, may contribute to the presence of Pb in TSP.

##### Vanadium (V)

Vanadium concentrations of TSP at Gisr Al Soltan site ranged from 0.033 µg/m^3^ to 0.070 µg/m^3^ and at the Mnaqbad site ranged from 0.029 µg/m^3^ to 0.298 µg/m^3^. The average Vanadium concentration in Assiut City (0.111 µg/m^3^) is below AQL (0.5 µg/m^3^) yet higher than in many other areas around the world. Vanadium, which is usually associated with fossil fuel combustion (especially heavy fuel oil), indicates that thermal power plants in Assiut City still depend on V-rich fuels.

From Table 12 and Figure 10, the concentrations of heavy metals in TSP have a decreasing order (Pb > Cr > V > Co > Ni > As > Cd), showing that lead and chromium as the dominant pollutants, assuring the need for air quality management and public health strategies in Assiut City, Egypt.

#### Health risk assessment

At high levels of heavy metals in total suspended particulates, humans are exposed to health risks via inhalation^18^. The high concentrations of different heavy metals (As, Cd, Co, Cr, Ni, Pb and V) in Table 12 are attributed to different localized pollution sources such as industrial activities and vehicles exhaust in the study area. These high values of air pollutants were cause for concern, hence, it was necessary to make health risk assessment. It is important to always make risk assessment transparent to the public with all the assumptions and parameters clearly stated. To assess carcinogenic and non-carcinogenic risks associated to the inhalation of TSP and deposited dust in the study area, cancer potency factor (CPF)^48^ and reference exposure level (REL)^49^^[,50^ for each heavy metal were attained, also, the estimated inhaled dose was calculated using the following simplified equation^51^.

2 $$\:Dose - inh\left( {\frac{{mg}}{{kg.day}}} \right) = \frac{{C_{{air\:}} \left( {\frac{{\mu \:g}}{{m^{3} }}} \right)\: \times \:\left( {\frac{{1\:mg}}{{1000\:\mu \:g}}} \right) \times \:Breathing\:rate\:\left( {\frac{{m^{3} }}{{day}}} \right)}}{{Body\:weight\left( {kg} \right)}}$$

The heavy metals concentrations ($\:\frac{\mu\:g}{{m}^{3}}$) in TSP samples were used to directly substitute $\:{C}_{air\:}$in the previous equation to calculate the dose inhaled by a human with average weight of 70 kg and average breathing rate of 20 $\:\frac{{m}^{3}}{day}$.

- Cancer risk assessment

The cancer risk values in Table 14 are the probability of developing cancer over a life time. The carcinogenic risk was obtained by multiplying each calculated inhalation dose ($\:\frac{mg}{kg.day}$) by the corresponding CPF $\:{\left(\frac{mg}{kg.day}\right)}^{-1}$ for each heavy metal. CPF describes the potential risk of developing cancer per unit of average Daily dose over a 70-year lifetime. According to U. S. EPA^52^, a risk range of $\:{1\times\:10}^{-6}$ to $\:{1\times\:10}^{-4}$ is considered acceptable. From Table 14 it was observed that the calculated cancer risk for As, Co and Cr exceeded this acceptable margin. Cr recorded the highest values of cancer risk ($\:2\times\:{10}^{-2}$) for TSP inhalation with expected 20,000 cancer cases per million of exposed individuals.

b. Non-cancer risk assessment

**Fig. 11:**
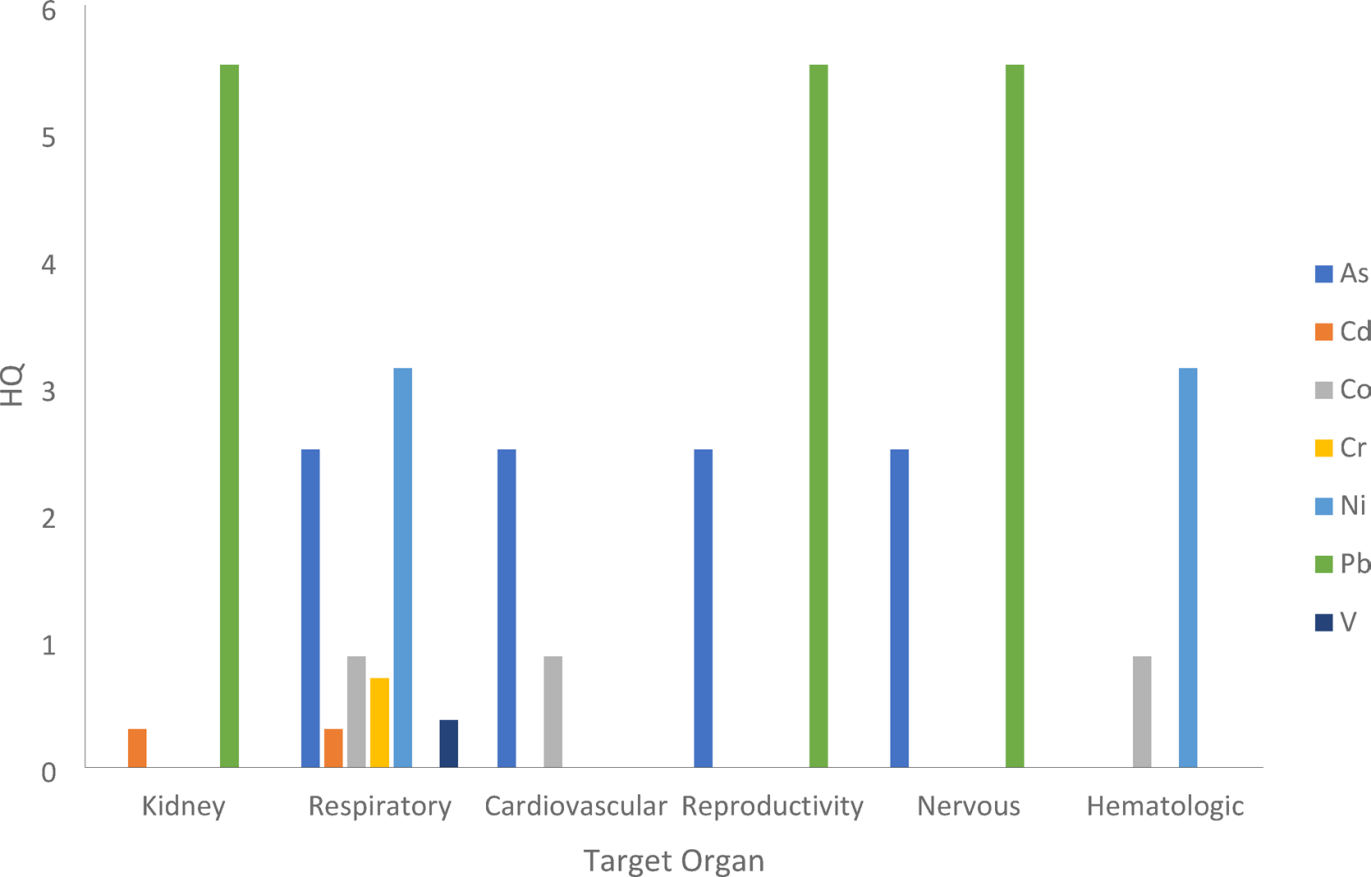
Hazard Quotient for different target organs based on inhalation dose of different heavy metals.

Non-carcenogenic risk is expressed by the hazard quotient (HQ) values in Table 13 and Figure 11. HQ are representing the ratio of the inhaled concentration to the REL for each heavy metal. Inhalation RELs are air concentrations or doses at or below which adverse noncancer health impacts are not expected. OEHHA/ARB approved chronic REL inhalation for different heavy metals (µg/m³) with different target organs, as listed in the Consolidated Table of OEHHA/ARB Approved Risk Assessment Health Values^49,50^.

According to U. S. EPA^52^, if HQ exceeds 1, it indicates a potential adverse health impact. From Table 13 it was observed that the calculated values of HQ for As, Ni and Pb are exceeding 1, highlighting a strong eventuality of non-carcinogenic risk for the population exposed to these pollutants in the study area. Pb recorded the highest HQ value (almost 6) for TSP inhalation which implies great helth concerns in the long-term exposure of this society.

**Fig. 12:**
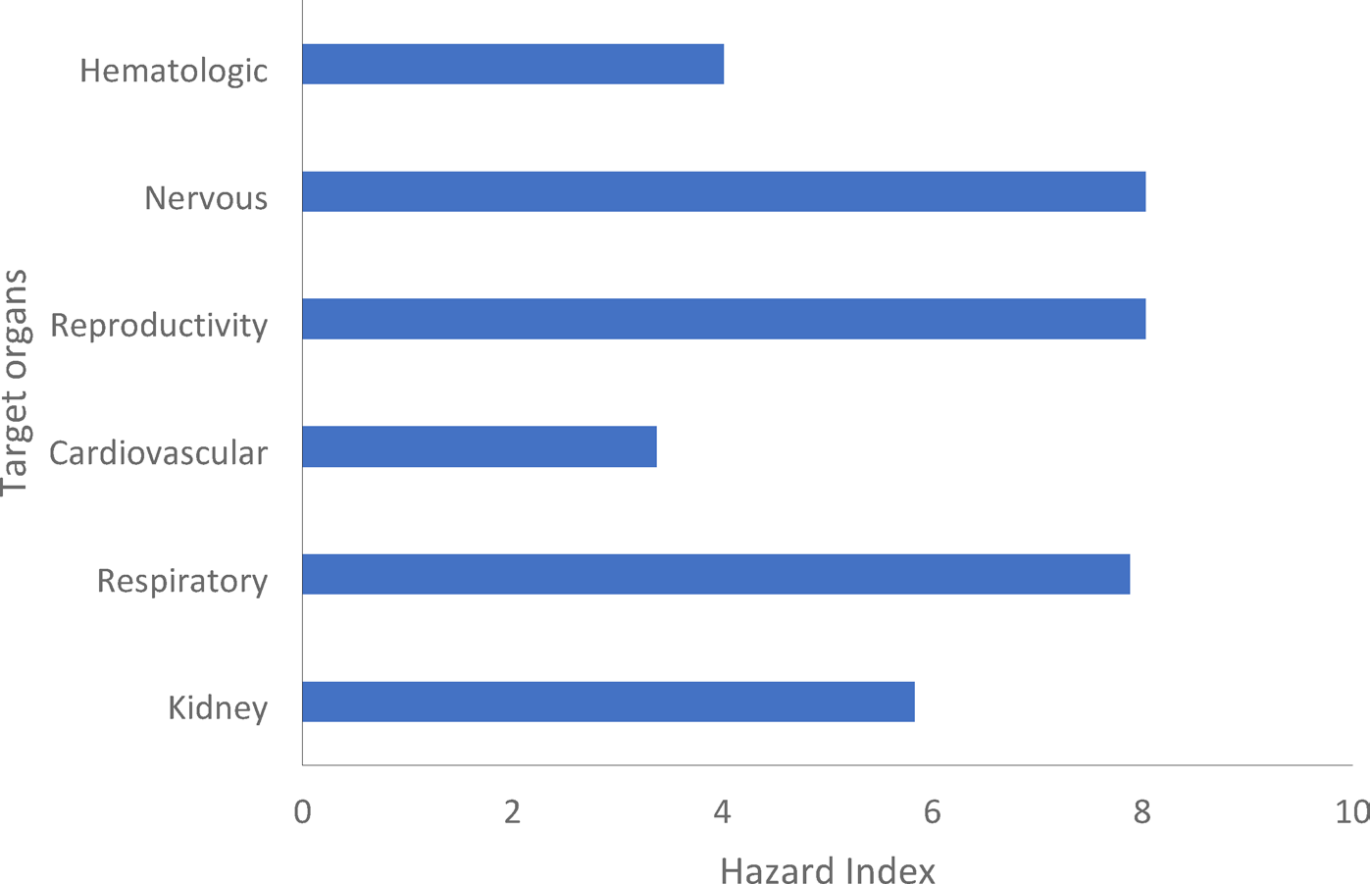
Hazard Index for different target organs based on hazard quotient of different heavy metals.

Moreover, the values of total Hazard indecies for each target organ $\:{HI}_{target\:organ}=\sum\:{HQ}_{all\:heavy\:metals}$ are illustrated in Table 14 and Figure 12. The high values of HI for different target organs due to inhalation of heavy metals bounded to TSP reveals great concerns of health risk in the society in the study area. The highest values of HI were observed for the most vulnerable target organs which are reproductive, nervous and respiratory systems. For reproductive and nervous systems, with HI of 8, it is clear that the major contributors is Arsenic and Lead. Additionally, as illustrated in Figure 11 and Table 14, Ni has a significant effect on the deterioration of the respiratory system besides multiple heavy metals including As, Cd, Co, Cr and V. Other target organs with high HI such as kidney and cardiovescular system are expected to examine severe adverse effects on the long-term exposure to these pollutants.

**Table 13:** Cancer and Non-Cancer health risks due to chronic exposure of heavy metals in the study area.

Pollutant	Concentration ($\:{\mu\:}{g}/{{m}}^{3}$)	Dose inhalation $\:\left(\frac{{m}{g}}{{k}{g}.{d}}\right)$	Cancer health risk assessment			Non-cancer health risk assessment		
			CPF inhalation $\:\left(\frac{mg}{kg.d}\right)$ ^−1^	Cancer risk	Cancer cases per million	REL Inhalation ($\:\mu\:g/{m}^{3}$)	HQ	Target organ
As	$\:0.038$	$\:0.1086\times\:{10}^{-4}$	$\:12$	$\:1.30\times\:{10}^{-4}$	$\:130$	$\:1.5\times\:{10}^{-2}$	$\:2.5$	Cardiovascular
								Reproductivity
								Nervous Syst.
								Respiratory
Cd	$\:0.006$	$\:0.171\times\:{10}^{-5}$	$\:15$	$\:25\times\:{10}^{-6}$	$\:25$	$\:2\times\:{10}^{-2}$	$\:0.3$	Kidney
								Respiratory
Co	$\:0.087$	$\:0.2486\times\:{10}^{-4}$	$\:27$	$\:6.71\times\:{10}^{-4}$	$\:671$	$\:0.1$	$\:0.87$	Respiratory
								Cardiovascular
								Hematological
Cr	$\:0.138$	$\:0.3943\times\:{10}^{-4}$	$\:5.1\times\:{10}^{2}$	$\:2.011\times\:{10}^{-2}$	$\:20110$	$\:2\times\:{10}^{-1}$	$\:0.7$	Respiratory
Ni	$\:0.044$	$\:0.1257\times\:{10}^{-4}$	$\:9.1\times\:{10}^{-1}$	$\:11.44\times\:{10}^{-6}$	$\:12$	$\:1.4\times\:{10}^{-2}$	$\:3.14$	Hematological
								Respiratory
Pb	$\:0.829$	$\:0.23686\times\:{10}^{-3}$	$\:4.2\times\:{10}^{-2}$	$\:10\times\:{10}^{-6}$	$\:10$	$\:0.15$	$\:5.53$	Nervous Syst.
V	$\:0.111$	$\:0.3171\times\:{10}^{-4}$				$\:0.3$	$\:0.37$	Respiratory

**Table 14:** Hazard indices for all target organs for chronic exposure to heavy metals in the study area.

Pollutant	Target organ	Kidney	Respiratory	Cardiovascular	Reproductivity	Nervous	Hematologic
As	$\:HQ$		$\:2.5$	$\:2.5$	$\:2.5$	$\:2.5$	
Cd		$\:0.3$	$\:0.3$				
Co			$\:0.87$	$\:0.87$			$\:0.87$
Cr			$\:0.7$				
Ni			$\:3.14$				$\:3.14$
Pb		$\:5.53$			$\:5.53$	$\:5.527$	
V			$\:0.37$				
Total hazard index$\:\sum \: HQ$		$\:5.83$	$\:7.88$	$\:3.37$	$\:8.03$	$\:8.03$	$\:4.01$

### Limitations and potential sources of bias

For more transparency and context of our results, it is important to specify the potential sources of bias and the confounding factors that could affect the results. For sampling site selection, the measurements of heavy metals were conducted in two urban locations (Gisr Al Soltan and Manqabad) in Assiut City. These sites may not fully represent the variation of heavy metals levels across the entire city. This might lead to underestimation or overestimation of heavy metals concentration in Assiut City; hence, the authors encourage future investigations to cover the total city in the future. Also, for heavy metals measurements, minor analytical errors might contribute to minor bias, especially at low concentrations, despite the care being taken in following the quality assurance protocols. Additionally, the minor atmospheric variability such as change in wind direction, speed and other meteorological conditions during sampling might have led to a minor bias in the measurements of heavy metals.

## Conclusions

The present study investigated the total suspended particulates (TSP) samples and deposited dust samples and the concentrations of heavy metals bound to them. The samples was collected from six locations (Al Hamraa, Al Arbaeen, Gisr Al Soltan, Al Wilidiyyah, Manqbad, and Al Burah) with consideration to different air pollution sources in the area. The mostly polluted sites (Mnaqbad and Gisr Al Soltan) had average TSP concentrations higher than the AQL. Additionally, the TVL was exceeded by the same most polluted locations. The high conentrations of heavy metals in the collected samples were cause of concern. The health risk assessment showed exceedance in expected cancer cases for specific heavy metals. The values of HQ and HI in this study indicates multi-organ toxicity, hence, there is a need for mitigation efforts to reduce emissions from pollution sources. Future studies are strongly encouraged to expand the findings of current research by taking more samples of total suspended particle (TSP) and deposited dust. Additionally, future studies should examine the long-term health effects of heavy metal exposure on local populations.

## Data Availability

The data that support the findings of this study are available from the corresponding author upon reasonable request.
